# Evaluating the Efficacy of ChatGPT as a Patient Education Tool in Prostate Cancer: Multimetric Assessment

**DOI:** 10.2196/55939

**Published:** 2024-08-14

**Authors:** Damien Gibson, Stuart Jackson, Ramesh Shanmugasundaram, Ishith Seth, Adrian Siu, Nariman Ahmadi, Jonathan Kam, Nicholas Mehan, Ruban Thanigasalam, Nicola Jeffery, Manish I Patel, Scott Leslie

**Affiliations:** 1 Department of Urology Saint George Hospital Kogarah Australia; 2 Faculty of Medicine The University of New South Wales Sydney Australia; 3 Surgical Outcomes Research Centre Sydney Australia; 4 Faculty of Medicine University of Sydney Sydney Australia; 5 Department of Surgery Peninsula Health Victoria Australia; 6 Concord Institute of Academic Surgery Concord Hospital Sydney Australia; 7 Department of Urology Chris O’Brien Lifehouse Sydney Australia; 8 Royal Prince Alfred Hospital Institute of Academic Surgery Royal Prince Alfred Hospital Sydney Australia; 9 Nepean Urology Research Group Nepean Hospital Sydney Australia; 10 Department of Urology Westmead Hospital Sydney Australia

**Keywords:** prostate cancer, patient education, large language model, ChatGPT, AI language model, multimetric assessment, artificial intelligence, AI, AI chatbots, health care professional, health care professionals, men, man, prostate, cancer, decision-making, prostate specific, antigen screening, medical information, natural language processing, NLP

## Abstract

**Background:**

Artificial intelligence (AI) chatbots, such as ChatGPT, have made significant progress. These chatbots, particularly popular among health care professionals and patients, are transforming patient education and disease experience with personalized information. Accurate, timely patient education is crucial for informed decision-making, especially regarding prostate-specific antigen screening and treatment options. However, the accuracy and reliability of AI chatbots’ medical information must be rigorously evaluated. Studies testing ChatGPT’s knowledge of prostate cancer are emerging, but there is a need for ongoing evaluation to ensure the quality and safety of information provided to patients.

**Objective:**

This study aims to evaluate the quality, accuracy, and readability of ChatGPT-4’s responses to common prostate cancer questions posed by patients.

**Methods:**

Overall, 8 questions were formulated with an inductive approach based on information topics in peer-reviewed literature and Google Trends data. Adapted versions of the Patient Education Materials Assessment Tool for AI (PEMAT-AI), Global Quality Score, and DISCERN-AI tools were used by 4 independent reviewers to assess the quality of the AI responses. The 8 AI outputs were judged by 7 expert urologists, using an assessment framework developed to assess accuracy, safety, appropriateness, actionability, and effectiveness. The AI responses’ readability was assessed using established algorithms (Flesch Reading Ease score, Gunning Fog Index, Flesch-Kincaid Grade Level, The Coleman-Liau Index, and Simple Measure of Gobbledygook [SMOG] Index). A brief tool (Reference Assessment AI [REF-AI]) was developed to analyze the references provided by AI outputs, assessing for reference hallucination, relevance, and quality of references.

**Results:**

The PEMAT-AI understandability score was very good (mean 79.44%, SD 10.44%), the DISCERN-AI rating was scored as “good” quality (mean 13.88, SD 0.93), and the Global Quality Score was high (mean 4.46/5, SD 0.50). Natural Language Assessment Tool for AI had pooled mean accuracy of 3.96 (SD 0.91), safety of 4.32 (SD 0.86), appropriateness of 4.45 (SD 0.81), actionability of 4.05 (SD 1.15), and effectiveness of 4.09 (SD 0.98). The readability algorithm consensus was “difficult to read” (Flesch Reading Ease score mean 45.97, SD 8.69; Gunning Fog Index mean 14.55, SD 4.79), averaging an 11th-grade reading level, equivalent to 15- to 17-year-olds (Flesch-Kincaid Grade Level mean 12.12, SD 4.34; The Coleman-Liau Index mean 12.75, SD 1.98; SMOG Index mean 11.06, SD 3.20). REF-AI identified 2 reference hallucinations, while the majority (28/30, 93%) of references appropriately supplemented the text. Most references (26/30, 86%) were from reputable government organizations, while a handful were direct citations from scientific literature.

**Conclusions:**

Our analysis found that ChatGPT-4 provides generally good responses to common prostate cancer queries, making it a potentially valuable tool for patient education in prostate cancer care. Objective quality assessment tools indicated that the natural language processing outputs were generally reliable and appropriate, but there is room for improvement.

## Introduction

Artificial intelligence (AI) chatbots have made significant strides in recent years [1]. This was emphatically signposted with the launch of ChatGPT-3 (OpenAI) [2] in November 2022, with ChatGPT becoming the most popular web-based tool for both patients and health care professionals [3,4]. Now in its fourth iteration (ChatGPT-4), the AI language model can generate responses to a wide range of health questions and topics [5]. AI chatbots, such as ChatGPT, have the potential to significantly impact patient education and disease experience by providing reliable, accessible, and personalized information [5,6]. One patient population that stands to benefit from this is men who are concerned about prostate cancer.

With the rising prevalence of prostate cancer globally—accounting for an estimated 1,414,259 new cases and over 375,304 deaths in 2020 alone—there is an urgent need for accurate and timely patient education information [7]. The rate of prostate cancer survivorship is increasing, but this comes with its own challenges such as escalating health care costs and large numbers of survivors requiring ongoing care [4]. In this context, shared decision-making becomes pivotal, particularly concerning prostate-specific antigen screening and prostate cancer treatment selection [4]. Given the various treatments available, management decisions can be greatly influenced by a patient’s understanding of the anatomical, functional, and psychological impacts of treatment [8]. Side effects, such as urinary incontinence and erectile dysfunction, can severely affect a patient’s quality of life, necessitating well-informed patients, and treatment choices [9]. Furthermore, patient education has been shown to minimize psychological impacts such as depression and treatment regret [10].

There are well-documented issues with unmet information needs of both men and their support networks throughout the prostate cancer care continuum [11]. This includes challenges related to information quality and readability [12]. The assessment of web-based health care information in prostate cancer has been well described through multiple domains including web page articles, YouTube (Google), and social media [11]. The internet is now often the first source of information for men (and their stakeholders) seeking answers about diagnosis, treatment, and prognosis [9]. Despite this trend, most long-term literature suggests that web-based health information is of moderate to poor quality [11-13].

AI chatbots are a potential solution to fill the prostate cancer information quality gap [3]. Given their scalability, AI chatbots can reach a wide demographic, including those in remote or underserved communities where medical resources are scarce [3]. Natural language processing technologies (NLPTs) enable these platforms to present complex jargon in patient-specific terms, with the potential to address eHealth literacy variability, and to enhance patient understanding [14]. Such platforms are also able to do this across a diverse number of languages [15]. Despite these qualities, the accuracy and reliability of AI chatbot medical information must still be assessed using rigorous evaluation tools. Only a handful of studies have begun to test ChatGPT’s applicability in prostate cancer: one testing its knowledge directly with questions and statements [16] and another assessing its appropriateness in screening recommendations [17]. However, a significant knowledge gap persists in understanding the quality and safety of information patients receive from ChatGPT-4 for common internet queries. Ongoing evaluation is a necessary step to build health care provider confidence in these new technologies while ensuring that patients have access to vetted and safe health care and educational information.

This study aims to demonstrate and assess the quality of ChatGPT responses to commonly asked patient education topics in prostate cancer care. By doing so, this study seeks to (1) illustrate to clinicians whether ChatGPT-4 is currently a reliable and safe patient education tool for prostate cancer information and (2) provide clinicians with a greater understanding of the current strengths and limitations of health-based queries which patients are likely to encounter when using technologies such as ChatGPT-4.

A range of assessment tools will be applied to the AI-generated responses to assess output quality, safety, understandability, actionability, ease of use, readability, and reliability. A parallel assessment of the outputs by prostate cancer experts will also be conducted.

## Methods

### Question or Keyword Strategy

Questions tested with the AI chatbot model (ChatGPT-4) were selected through an iterative process of literature and Google Keyword analysis. Literature concerning the information needs of men considering prostate cancer investigation and treatment was reviewed to determine the most common information topics and prostate cancer questions of interest to men [11,18-21]. Subsequently, worldwide Google Trends data were analyzed to provide a more current public measure of prostate cancer information searches [22]. Using “prostate cancer” as a keyword, both rising and top “related topics” and “related queries” of the past year were collected. Finally, while limited to training materials up to 2021, ChatGPT was itself queried, asking “What are the most common prostate cancer questions asked to ChatGPT?” (Multimedia Appendix 1). The 2 authors thematically analyzed this information to define the following eight questions to discuss with the AI model: (1) What are the symptoms of prostate cancer? (2) What are the risk factors for prostate cancer? (3) What is the survival rate of prostate cancer? (4) How is prostate cancer diagnosed? (5) What age should men start getting screened for prostate cancer? (6) What are the pros and cons of treatment options for prostate cancer? (7) How does prostate cancer affect sexual function? and (8) How does prostate cancer affect bladder function?

Each question was posed to ChatGPT-4 with an additional request for references. A new ChatGPT account was established with a novel email address for each prompt in an effort to reduce the potential effects of each response on subsequent outputs of the AI model. Each output was recorded for individual quality and readability assessment (Multimedia Appendix 2).

### Quality Assessment

#### Overview

Due to the current absence of tools to evaluate the quality of AI natural language outputs, each conversation was evaluated using modified versions of pre-existing information quality assessment tools. These included the Patient Education Materials Assessment Tool (PEMAT) and DISCERN criteria [23,24]. These tools were iteratively modified to accommodate the text-only nature and characteristics of AI natural language outputs. While DISCERN criteria have been adapted in literature, the PEMAT modification is new [25]. Internal validity testing was undertaken by 4 reviewers using ChatGPT outputs from similar question sets for breast cancer and bowel cancer. The reliability of each tool tested was satisfactory, with Cronbach α>0.8 (DISCERN 0.852, PEMAT 0.82, and Global Quality Score [GQS] 0.85). The GQS was not modified [26].

#### PEMAT-AI Tool

The PEMAT tool evaluates and compares the understandability and actionability of patient education materials [24]. The tool incorporates 17 items measuring understandability and 7 assessing actionability; these were reduced to 8 and 3, respectively, to suit the AI text-only outputs (Multimedia Appendix 3). Each item was given a single point if the criteria were met, and the total score was measured as a total percentage. Final scores were recorded as “pass” or “fail” based on the ≥70% cut‐off score set by the PEMAT guidelines [24].

#### DISCERN-AI Tool

The DISCERN criteria is a previously validated tool that aids health care consumers and health practitioners in appraising the quality of health care treatment information [23,24]. To address the AI output, these criteria were modified to 7 questions (of the original 15) on a scale of 1 to 3, using questions 3-9 (Multimedia Appendix 4). Based on previous DISCERN quality assessment in the literature, each output was scored as very poor (6), poor (7-9), fair (8-12), good (13-15), and excellent (16-18) quality patient education material [27,28].

#### GQS Tool

The GQS is a 5-point Likert scale based on the quality of information, and the flow and ease of use of information presented via the web. The GQS encompasses a scale of 1 to 5; where 1 indicates “low quality” and 5 implies “high quality.” Results that received a score of 4 or 5 were rated high quality, those with a score of 3 were assessed as medium quality, and the ones with a score of 1 or 2 were categorized as low quality [25,29].

### Readability

The readability of the AI responses was assessed using a battery of established algorithms: the Flesch Reading Ease score, Gunning Fog Index, Flesch-Kincaid Grade Level, Coleman-Liau Index, and Simple Measure of Gobbledygook (SMOG) Index [30-33]. Multiple tools were used in an effort to limit the bias of each respective algorithm [34]. Each AI output text was copied to Microsoft Word to maintain formatting, and then to Readable.com for analysis [35,36]. Results from the answered questions were averaged across all outputs into a readability consensus [37]. The Flesch Reading Ease score gauges text simplicity, where the score ranges from 0 to 100, with higher scores indicating easier readability. Texts with a score between 60 and 70 are generally considered to be at an eighth- to ninth-grade reading level and are usually easier for the average adult to read. The Gunning Fog Index and The Flesch-Kincaid Grade Level measure sentence complexity, the score represents the number of years of formal education a reader would need to understand the text on the first reading. For example, a score of 12 would mean the text is suitable for a 12th-grade reading level or higher. The Coleman-Liau Index is similar to Gunning Fog and The Flesch-Kincaid Grade Level but focuses on character count. This score also correlates with a US school grade level but is calculated using the number of characters instead of syllables, making it more suited for languages where syllable count is less indicative of complexity. The SMOG Index evaluates syllable density to assess readability and is often used for checking health messages. A score of 12 would mean the text is suitable for someone with at least a 12th-grade level of reading comprehension.

### Natural Language Assessment Tool for AI

Expert review of each output was undertaken by 7 independent experienced urologists, using an assessment framework (Natural Language Assessment Tool for AI [NLAT- AI]) developed to assess the accuracy, safety, appropriateness, actionability, and effectiveness of information. Each domain was scored on a 5-point Likert scale (1=strongly disagree, to 5=“strongly agree”; Multimedia Appendix 5). All results were collated and presented as descriptive statistics. Qualitative feedback on each domain was sought regarding potential improvement and overall performance.

### References Assessment

Due to known issues of AI hallucination: “the phenomenon of a machine, such as a chatbot, generating seemingly realistic sensory experiences that do not correspond to any real-world input,” a final brief tool (Reference Assessment AI [REF-AI]) was developed for analysis of the references provided by AI outputs [38]. Each reference was reviewed by accessing the content via the direct link provided by the AI output, or a Google search of the reference. This tool assessed for reference hallucination (real or not), relevance (correlation between the references and AI output), and quality of references (type of institution linked to the reference). Each criterion was assessed with a score of 1-3, with a lower summative score indicating lower reference quality, and a higher score indicating high reference quality (Multimedia Appendix 6). Scores were averaged to yield a composite score for each axis of evaluation. The reliability of this tool tested similar question sets for breast cancer and bowel cancer was satisfactory (0.81).

### Ethical Considerations

After consultation with the local institutional review board, it was determined that no formal ethical approval was required for this study as no human or animal participants were involved.

## Results

### ChatGPT Outputs

**Figure 1:**
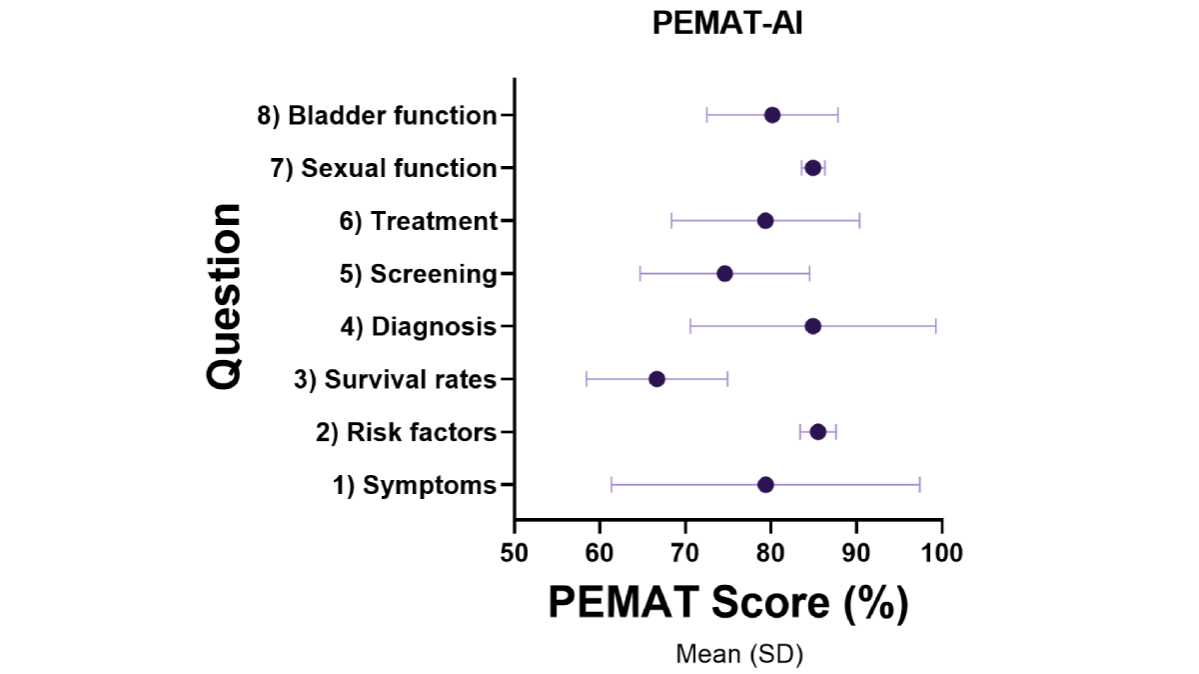
PEMAT-AI mean score by ChatGPT question output. PEMAT-AI: Patient Education Materials Assessment Tool for Artificial Intelligence.

**Figure 2:**
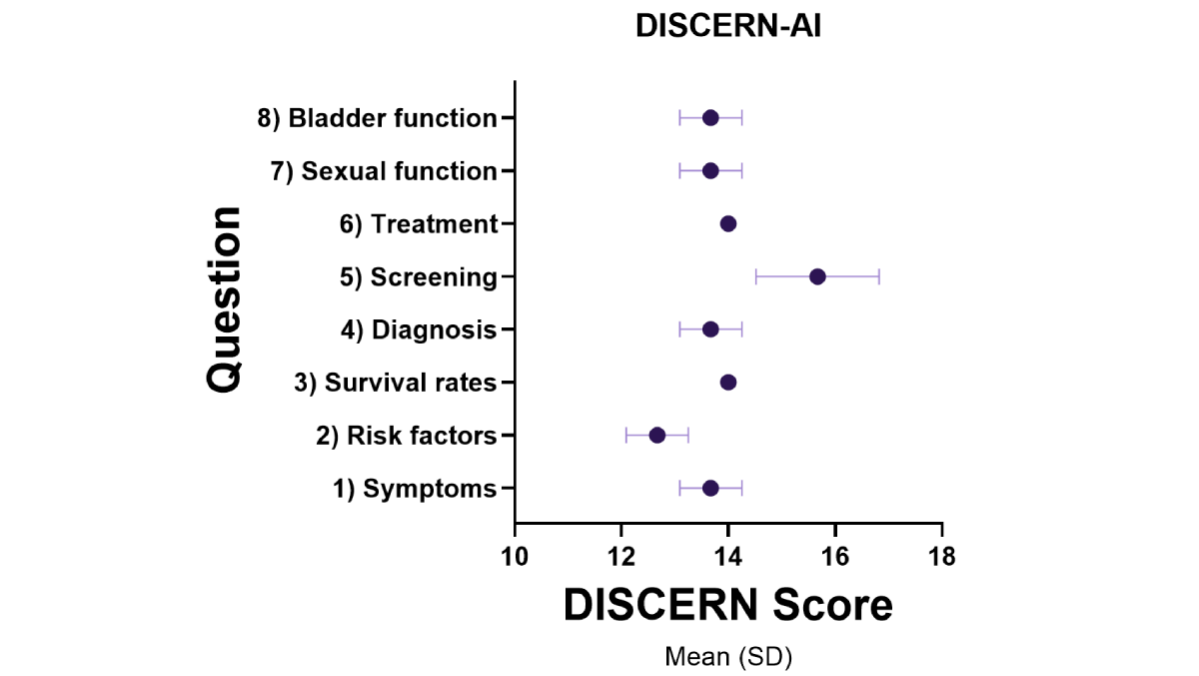
DISCERN-AI mean score by ChatGPT question output. AI: artificial intelligence.

**Figure 3:**
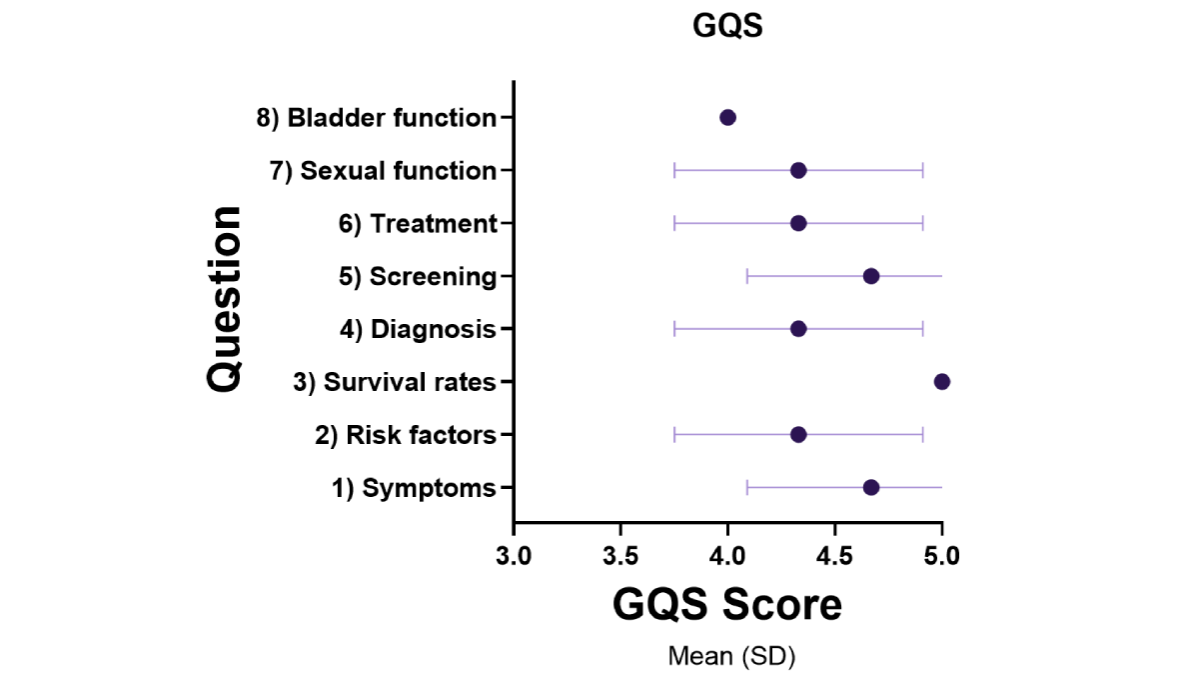
GQS mean score by ChatGPT question output. GQS: Global Quality Score.

The responses generated by the AI model, ChatGPT-4, provided broad, medically aligned information (Multimedia Appendix 2). The assessment of the ChatGPT-4 output using PEMAT-AI, DISCERN-AI, and GQS patient education material assessment tools demonstrated high results across all tools. The pooled PEMAT-AI understandability score easily passed the acceptability threshold of >70% (mean 79.44%, SD 10.44%); only question 3 failed the >70% threshold at 66.67% while the remaining were 76% or greater (Figure 1). The pooled DISCERN-AI rating was scored as “good” quality 77% (mean 13.88, SD 0.93), and all individual questions rated “good” on the DISCERN-AI except for question 5, which scored excellent (mean 15.67; Figure 2). The pooled GQS was rated as high (mean 4.46, SD 0.50 out of 5; Figure 3). Assessment tool results for each question are tabulated and graphed (Table 1 and Figures 1-3). Reliability testing was high with Cronbach α=0.846.

**Table 1:** Quality assessment tools.

Assessment			PEMAT-AI^a^, mean (SD)		DISCERN-AI, mean (SD)		GQS^b^, mean (SD)
**Questions**							
	Symptoms	79.37 (18.03)		13.67 (0.58)		4.67 (0.58)	
	Risk factors	85.51 (2.10)		12.67 (0.58)		4.33 (0.58)	
	Survival rates	66.67 (8.25)		14.00 (0.00)		5.00 (0.00)	
	Diagnosis	84.92 (14.35)		13.67 (0.58)		4.33 (0.58)	
	Screening	74.60 (9.91)		15.67 (1.15)		4.67 (0.58)	
	Treatment	79.36 (10.99)		14.00 (0.00)		4.33 (0.58)	
	Sexual function	84.92 (1.37)		13.67 (0.58)		4.33 (0.58)	
	Bladder function	80.16 (7.65)		13.67 (0.58)		4.00 (0.00)	
Total			79.44 (10.44)		13.88 (0.93)		4.46 (0.50)

^a^PEMAT-AI: Patient Education Materials Assessment Tool for Artificial Intelligence.

^b^GQS: Global Quality Score.

### NLAT-AI Assessment

**Figure 4:**
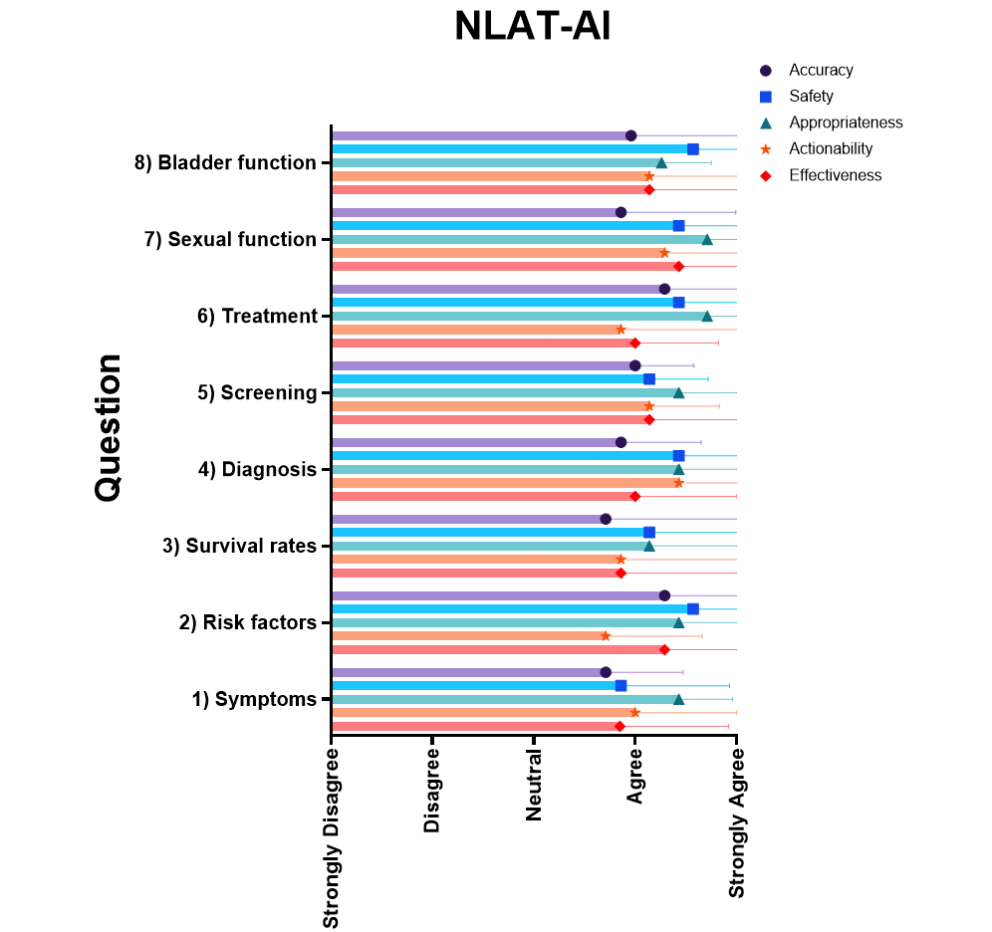
NLAT-AI mean score by ChatGPT question output. NLAT-AI: Natural Language Assessment Tool for Artificial Intelligence.

Expert assessment of the AI outputs with NLAT-AI was consistent with a mean >3.0 out of 5.0 (neutral) in all domains across all question replies. NLAT-AI pooled means included accuracy of 3.96 (SD 0.91), safety of 4.32 (SD 0.86), appropriateness of 4.45 (SD 0.81), actionability of 4.05 (SD 1.15), and effectiveness of 4.09 (SD 0.98). Descriptive statistics for each question are tabulated and graphed (Table 2 and Figure 4). Internal validity testing demonstrated high reliability with Cronbach α=0.906.

Qualitative feedback via NLAT-AI on questions 1 through 8 indicates some areas for improvement despite the generally accurate and easy-to-understand nature of responses. Common themes were a need for greater specificity, updated and comprehensive information, and a more globally inclusive perspective (Textbox 1). Outputs were often characterized as good starting points or overviews which could benefit patients.

**Table 2:** Natural Language Assessment Tool for Artificial Intelligence assessment.

Assessment			Accuracy, mean (SD)		Safety, mean (SD)		Appropriateness, mean (SD)	Actionability, mean (SD)		Effectiveness, mean (SD)
**Questions**										
	Symptoms	3.71 (0.76)		3.86 (1.07)		4.43 (0.53)		4.00 (1.00)	3.85 (1.07)	
	Risk factors	4.29 (0.76)		4.57 (0.53)		4.43 (0.98)		3.71 (0.95)	4.29 (0.76)	
	Survival rates	3.71 (1.60)		4.14 (1.60)		4.14 (1.07)		3.86 (1.68)	3.86 (1.68)	
	Diagnosis	3.86 (1.07)		4.43 (0.79)		4.43 (0.79)		4.43 (1.51)	4.00 (1.00)	
	Screening	4.00 (0.58)		4.14 (0.58)		4.43 (0.79)		4.14 (0.69)	4.14 (0.90)	
	Treatment	4.29 (0.49)		4.43 (0.79)		4.71 (0.49)		3.86 (1.46)	4.00 (0.82)	
	Sexual function	4.00 (0.82)		4.43 (1.13)		4.71 (0.49)		4.29 (0.79)	4.43 (0.79)	
	Bladder function	3.86 (1.07)		4.57 (1.07)		4.26 (0.49)		4.14 (0.90)	4.14 (0.90)	
Total			3.96 (0.91)		4.32 (0.86)		4.45 (0.81)	4.05 (1.15)		4.09 (0.98)

**Natural Language Assessment Tool for Artificial Intelligence qualitative feedback.**

**Question 1: Symptoms**

- Overall, a reasonable answer to the question... more emphasis should be put on the fact that prostate cancer is usually asymptomatic, usually detected on screening, only symptomatic when advanced.
- Could have been better if discussed symptoms of locally advanced prostate cancer (LUTS, haematuria, etc) and symptoms of metastatic prostate cancer (bone pain, weight loss, etc)
- Need to strongly emphasize that most prostate cancers are asymptomatic so prostate-specific antigen testing is necessary

**Question 2: Risk factors**

- Considering that this is tailored for Americans, it may not be actionable for others.
- From a safety perspective, I would emphasize the importance of seeking medical review in the event of family history.
- Remove the modifiable risk factors as it makes patients think they can prevent it

**Question 3: Survival rates**

- There is...no mention of the impact of treatment on survival so a patient could be forgiven for thinking this was survival rates in the event of no treatment being given.
- “Relative survival” is not clearly explained.
- The survival rate [is] overestimated in organ-confined disease as this is far more complex. It should be more clarified.
- When talking about prostate cancer survival 10 years is the minimum that should be discussed
- Fairly good- this is what I would tell my patients.

**Question 4: Diagnosis**

- Overall reasonable answer from ChatGPT
- CT and bone scans are used for staging; but now in Australia is superseded by PSMA
- Reasonable answer. Some inaccuracies in how the tests are used, as well as their sequencing. PSMA PET not mentioned which is an important part of diagnosis and staging. These deficiencies likely reflect the rapidly evolving nature of prostate cancer diagnosis.
- The answer is easy to understand and general principles of diagnosis sound.

**Question 5: Screening**

- Point 2 is very contentious and...gives a very one-sided view of prostate cancer screening.
- This is only appropriate for American audience.
- Point 2 is concerning as this represents one [clinician] group who is very much against prostate cancer screening... therefore may risk not giving a balanced view.
- No mention of any local guidelines, and no EAU [European Association of Urology] guidelines.

**Question 6: Treatment**

- Very useful summary for patients immediately after diagnosis.
- No mention of novel tx [treatments] eg: focal therapy, cryo, HIFU
- No mention of robotic surgery versus open surgery
- This is a very simple table about the pros and cons.

**Question 7: Sexual function**

- Overall a very good answer—misses minor points
- Very well written
- Would also mention that erectile function improves over time.
- Surgery does not damage the vessels for erection

**Question 8: Bladder function**

- Nice summary
- Accurate and easy to understand
- Minor issues only with the discussion on stress or urge incontinence
- Hormone therapy should not causes bladder dysfunction. In fact, it might improve it

### Readability Assessment

The readability algorithm consensus was “difficult to read” (Flesch Reading Ease score mean 45.97, SD 8.69; Gunning Fog Index mean 14.55, SD 4.79), averaging an 11th-grade reading level, equivalent to 15- to 17-year-olds (Flesch-Kincaid Grade Level mean 12.12, SD 4.34; The Coleman-Liau Index mean 12.75, SD 1.98; SMOG Index mean 11.06, SD 3.20). Questions 1 and 2 were the easiest to read scoring an 8th-grade level, while questions 6 (grade 23 level), 7 (grade 12 level), and 8 (grade 13 level) were very difficult to read (Table 3).

**Table 3:** Readability assessment.

Assessment		Flesch Reading Ease score	Gunning Fog Index	Flesch-Kincaid Grade Level	The Coleman-Liau Index	SMOG^a^ Index
**Questions**						
	Symptoms	53.2	10.1	8.7	11	7.8
	Risk factors	59.4	8.5	7.7	11	7.6
	Survival rates	51.4	15	11	10	11.1
	Diagnosis	46.2	13.4	10.9	13	10.3
	Screening	49.3	13.7	11.1	12	10.6
	Treatment	57.2	25.7	22.8	16	18.7
	Sexual function	39.9	14.9	11.6	14	11
	Bladder function	31.2	15.1	13.2	15	11.4
Pooled total		45.97	14.55	12.12	12.75	11.06

^a^SMOG: Simple Measure of Gobbledygook.

### REF-AI Assessment

REF-AI identified 2 reference hallucinations from 30 total references across all questions (pooled REF-AI Real mean 2.86). Most references effectively supported the text, while 4 questions had 1 or 2 citations that did not directly support the information provided (Table 4; pooled REF-AI supporting mean 2.75). A total of 86% (26/30) of references were from reputable government organizations, while 2 were direct citations from scientific literature (pooled REF-AI source mean 2.13). Individual statements were provided a direct reference in only 3 outputs. The remaining outputs instead provided a list of references at the bottom of the text. Some direct links to references were not complete, instead delivering the user to the organization’s primary website URL, likely reflecting updated website directories since the 2021 ChatGPT indexation. The 2 hallucinated references were present in questions 7 and 8, where weblinks did not connect and despite extensive Google and library searches, the original material was unable to be located.

**Table 4:** Reference Assessment Artificial Intelligence assessment.

Assessment			Real, mean (SD)		Supporting, mean (SD)	Source, mean (SD)
**Questions**						
	Symptoms	3.00 (0.00)		3.00 (0.00)		2.00 (0.00)
	Risk factors	3.00 (0.00)		2.67 (0.58)		2.00 (0.00)
	Survival rates	3.00 (0.00)		2.67 (0.58)		2.00 (0.00)
	Diagnosis	3.00 (0.00)		3.00 (0.00)		2.00 (0.00)
	Screening	3.00 (0.00)		3.00 (0.00)		3.00 (0.00)
	Treatment	3.00 (0.00)		3.00 (0.00)		2.00 (0.00)
	Sexual function	3.00 (0.00)		2.33 (0.58)		2.00 (0.00)
	Bladder function	2.00 (0.00)		2.33 (0.58)		2.00 (0.00)
Total			2.86 (0.00)		2.75 (0.29)	2.13 (0.00)

## Discussion

### Principal Findings

In the digital information age, understanding what patient health information is accessed and the quality of this information is crucial. This study demonstrates several examples of information that patients (and their caregivers) may encounter when conducting searches related to prostate cancer management. In our analysis, ChatGPT-4 provided generally comprehensive answers to prostate cancer questions, mostly in line with current medical guidelines and literature. ChatGPT-4 demonstrated promise when assessed with a range of patient education and information quality assessment tools, as well as expert review. Robust scores and expert feedback indicate that the generated content was reliable, safe, and actionable for patients, albeit with room for improvement in minor nuanced details, global applicability, and readability.

Current evidence indicates that 75% of people turn to the internet for decision-making during a health crisis [39]. Despite the abundance of available patient information, studies assessing the quality of digital health information indicate significant shortcomings [40]. For prostate cancer, the quality of information that reaches the patient is known to be inconsistent [11,20,41-43]. For example, a previous assessment of the top 100 “prostate cancer” web page results identified via search engine query showed that only 11.1% of sites demonstrate an excellent on the original DISCERN criteria [11]. While our analysis has used necessarily disparate methods, a comparison of our DISCERN-AI results (good-excellent) to static web page DISCERN scores suggests that ChatGPT prostate cancer information outputs may be of a higher quality than many traditional web pages [11]. ChatGPT4 appears capable of providing broad and largely accurate information which may further augment self-directed patient or stakeholder enquiry. Nevertheless, a direct comparison of ChatGPT outputs to established gold standard information sources is necessary to clearly define the role of this new communication technology as part of patient care and education.

Our findings appear to differ from Coskun et al [16], where ChatGPT-3 had accuracy issues using queries generated by the European Association of Urology Patient Information. Interestingly, Zheng et al [17] discovered that ChatGPT-4 can offer suitable counseling on disease prevention and screening for prostate cancer patients. These differences may represent the rapid evolution of the algorithm as our testing used the newer model. Exclusive use of US-centric guidelines raised questions of bias among our experts. Others have also highlighted such bias, noting that 51% of training data for major large language models is US sourced [44,45]. The disparities between ChatGPT-3 and ChatGPT-4 highlight the continual advancement and refinement of the underlying technology, reinforcing the need for periodic assessment and validation as newer models emerge [5,17]. Conversely, a lack of validated and reproducible tools to make reliable quality assessments of NPLTs is likely to play a role in varied results within this juvenile domain of clinical research [46]. While the methods used in our study were an effort to standardize output assessment in our work, we recognize and encourage further rigorous work to develop validated and reproducible assessment tools that can be applied to a range of NPLT outputs and platforms.

Despite the NLAT-AI rating, and general appropriateness of the language across all questions, the objective readability from algorithms demonstrated a high reading level and difficulty. This is likely reflective of the literacy bias present among our highly educated expert pool [34,47,48]. While the recommended reading level for patient education material varies between organizations, the consensus is that it should generally lie between grade 6 and 8 reading levels [47,49]. The readability algorithms thus suggest that the generated content may be challenging for some readers. These findings are of importance given that lower readability may limit accessibility for certain socioeconomic or minority groups [47]. Literacy is a known negative correlate of prostate cancer health outcomes [9,50-52]. Compounding this concern is the effect of the user’s overarching eHealth literacy, which is likely to affect chatbot engagement behaviors and patterns of information comprehension and use [3,16,50,53]. Effects of both traditional literacy and eHealth literacy on the end user experience of NLPTs require urgent investigation due to the pervasiveness that these technologies are already presenting within society and in web-based health communication [54,55].

The digital nature of the ChatGPT-4 model, where users can continuously engage and seek clarifications, offers a potential advantage and solution to static patient information materials. Although beyond this study’s scope, the ChatGPT-4 model permits ongoing discussions, enabling patients to seek clarifications of information. These conversations allow for personalized explanations related to patient health results, the opportunity to simplify language, and may ultimately address some concerns raised by our expert assessors. This is an extremely powerful and unique component of this new digital technology. Future iterations of such models may benefit by incorporating clear adaptability features, where the complexity and specificity of the content can be adjusted based on user preferences or needs. Further studies are required to explore how the longitudinal and dynamic features of NLPTs affect information quality and patient comprehension. This will be particularly important in comparison to traditional website and social media-based information sources which currently dominate the landscape of self-educative information sourcing in prostate cancer care [11,20,56]. NPLTs with predetermined or flexible user settings attuned to patient preference, needs, or literacy level are a potential futurist pathway to cost-effective and scalable forms of tailored patient health education materials.

Hallucination, where information is fabricated by the NLPT and presented as valid, is a well-documented phenomenon specific to NLPTs and ChatGPT [38]. This study demonstrated that hallucinations could occur when searching for prostate cancer with NPLT or chatbots. While only occurring in 2 instances of 30, these findings continue. Designation between hallucination and faux hallucination should also be considered. Faux-hallucination results from modified references after ChatGPT-4’s indexation, leading to broken links or lost references. Website redesign or content that no longer exists after the 2021 indexation is a potential etiology for hallucination that has not been fully explored. Equally, such disappearance of content with time may also match the definition of hallucination in the future. While not a prominent issue in this study, these findings continue to demonstrate the potential for fabricated information, which can be easily overlooked by the unassuming clinician, patient, or researcher. While still in its infancy, large language models must continue to solve the issue of hallucination before integration into high-risk systems, such as health care, can be considered.

While hallucinations are a notable concern, there are several other limitations of current NPLTs that need to be considered. Despite malleability, it is unknown whether the ChatGPT-4 model may fully replicate the nuance of human communication necessary for effective patient health education [3]. Additionally, the most significant limitation of ChatGPT is its potential for biased, outdated, or misleading content generation [1,4,6,53]. Even with relatively high-quality scores, this study shows that ChatGPT can still produce misleading or biased content under discriminatory and expert scrutiny, posing some element of risk for those with poor eHealth literacy [4,6,53]. However, while expert reviewers identified minor inaccuracies, none of these points were considered to be significantly concerning safety issues. Nevertheless, there is currently a lack of evidence to predict the impact of these technologies on patients’ understanding, decision-making, or health, without further inquiry and consideration of patients’ ability to interact with these new eHealth technologies. We strongly recommend clinicians report these concerns to prostate cancer patients and their stakeholders when guiding patient use of web-based information in their care. Furthermore, the opaque and dynamic nature of this technology’s private enterprise proprietary algorithms is also a concern [3,4,46]. Algorithm development will likely outpace quality assurance efforts and raise questions about the necessity of clinician involvement in NPLT model development that aims to present health-based information [3,45]. The effectively unknown and vast array of sources from which ChatGPT’s training data are derived raises ethical concerns. Without knowing the origins and credibility of such data, it is difficult for clinicians to fully trust generated content, presenting us with a modernized but perpetual issue of distrust in web-based information which may ultimately hinder adoption and progress [3,6,53]. Finally, there are also financial considerations; the cost of using ChatGPT-4 (as opposed to the currently free ChatGPT-3.5) or other NPLTs may form a barrier to widespread adoption in health care settings and has the potential to drive disparate levels of health care if not effectively managed and regulated.

### Limitations

Limitations of this study include the sample size of assessors, which may skew the evaluation of the included tool’s reliability and efficacy. The qualitative assessments of experts are at inherent risk of bias for or against the use of novel technology and ChatGPT-4. However, these experts are also deeply aware of the nature and quality of current prostate cancer education materials, providing additional insight that is of value to this work.

It is important to note this assessment was purposefully narrow in scope and may not reflect the myriad of interactions under the vast topics of prostate cancer. It is unknown how applicable these interactions are in wider prostate cancer education scenarios and ongoing investigation is required. Work is currently underway to assess an expanded question set with a comparison to currently accepted patient education gold standards in prostate cancer.

While not an explicit purpose of this study, the exploratory assessments used in this work (DISCERN-AI, PEMAT-AI, NLAT-AI, and REF-AI) demonstrate interreliability and replicability across several cancer-type information outputs. They may thus have potential use for clinicians and researchers interested in reviewing the quality of other cancer-based outputs of ChatGPT-4 or other NPLTs. Nevertheless, their validity requires further testing and greater investigation is necessary to develop specific tools to assess NPLT output quality in the long term.

### Conclusion

Our analysis found ChatGPT-4’s responses to common prostate cancer queries were of good quality, and a potentially useful patient education adjunct for prostate cancer care. Objective quality assessment tools were reflective of NPLT outputs, which were generally reliable and appropriate, although with room for improvement. Our expert panel was impressed by the appropriateness and safety of the language and information given. However, clinicians should be aware that there are several limitations to ChatGPT-4 prostate cancer outputs including hallucination, specificity issues, and difficult readability. Future studies are required to assess whether more longitudinal (back-and-forth) ChatGPT-4 discourse may offset some of the concerns highlighted in this analysis, and how patients of differing eHealth literacy levels may engage with and have care affected by such technologies.

## Appendix

Multimedia Appendix 1 Common prostate cancer questions.

Multimedia Appendix 2 ChatGPT-4 output.

Multimedia Appendix 3 Patient Education Materials Assessment Tool for AI (PEMAT-AI) tool.

Multimedia Appendix 4 DISCERN-AI (artificial intelligence) tool.

Multimedia Appendix 5 Natural Language Assessment Tool for Artificial Intelligence (NLAT-AI).

Multimedia Appendix 6 Reference Assessment Artificial Intelligence (REF-AI) tool.

## Abbreviations

AI: artificial intelligence
GQS: Global Quality Score
NLAT: Natural Language Assessment Tool
NLPT: natural language processing technology
PEMAT: Patient Education Materials Assessment Tool
REF-AI: Reference Assessment Artificial Intelligence
SMOG: Simple Measure of Gobbledygook
