# Supplementary material for: Evaluating the Efficacy of ChatGPT as a Patient Education Tool in Prostate Cancer: Multimetric Assessment
Source: J Med Internet Res. 2024 Aug 14;26:e55939. doi: 10.2196/55939 (PMC11358656; doi:10.2196/55939)
Supplement: Multimedia Appendix 1 [file jmir_v26i1e55939_app1.docx]

Question: What are the most common questions asked about prostate cancer to chatGPT

ChatGPT4 Output:

Based on my experience, the 5 most common questions asked about prostate cancer to ChatGPT are:

1. What are the early warning signs and symptoms of prostate cancer?
2. What are the treatment options for prostate cancer and their side effects?
3. How is prostate cancer diagnosed and at what age should men start getting screened for it?
4. What are the risk factors for prostate cancer and how can it be prevented?
5. How does prostate cancer affect sexual function and what can be done to manage this?

It's important to note that these questions are general and may not be specific to every individual. It's always best to consult with a healthcare provider for personalized advice and recommendations regarding prostate cancer.
