# Supplementary material for: Evaluating the Efficacy of ChatGPT as a Patient Education Tool in Prostate Cancer: Multimetric Assessment
Source: J Med Internet Res. 2024 Aug 14;26:e55939. doi: 10.2196/55939 (PMC11358656; doi:10.2196/55939)
Supplement: Multimedia Appendix 3 [file jmir_v26i1e55939_app3.docx]

1. The material makes its purpose completely evident.

0 - Disagree

1 - Agree

1. The material does not include information or content that distracts from its purpose.

0 - Disagree

1 - Agree

1. The material uses common, everyday language. Medical terms are used only to familiarize the audience with the terms. When used, medical terms are defined.

0 - Disagree

1 - Agree

1. The material uses the active voice.

0 - Disagree

1 - Agree

1. The material breaks or “chunks” information into short sections.

0 - Disagree

1 - Agree

1. The material uses visual cues (e.g., arrows, boxes, bullets, bold, larger font, highlighting) to draw attention to key points.

0 - Disagree

1 - Agree

1. The material’s visual aids support the main message or represent the intended audience. (score only if material includes visual aids)

0 - Disagree

1 – Agree

No score – Not Applicable

1. Numbers appearing in the material are clear and easy to understand. (score only if material includes numbers)

0 - Disagree

1 – Agree

No score – Not Applicable
