# Supplementary material for: Evaluating the Efficacy of ChatGPT as a Patient Education Tool in Prostate Cancer: Multimetric Assessment
Source: J Med Internet Res. 2024 Aug 14;26:e55939. doi: 10.2196/55939 (PMC11358656; doi:10.2196/55939)
Supplement: Multimedia Appendix 4 [file jmir_v26i1e55939_app4.docx]

1. **Is the information in the AI output relevant?**
   1. No - the information is not at all relevant.
   2. Partially - the information is relevant to some extent.
   3. Yes - the information is relevant.
2. **Is it clear what sources of information were used to compile the AI output?**
   1. No - no sources of evidence for the information are mentioned.
   2. Partially - the sources of evidence are clear to some extent.
      1. Give a this rating if one of the following hints is fulfilled:
         - A main statement or ‘fact’ is accompanied by a reference/source of evidence in the output [e.g. ‘Treatment using X has been found to be successful (source)]
         - A source of evidence is listed in a bibliography/reference list at the end of the output, or inserted as a hyperlink to another online publication or organisation.
      2. You may also give a partial rating to a publication which quotes a reference in the text for SOME but not ALL of the main statements or ‘facts’, although you will need to use your judgement to decide when a reference would be expected.
   3. Yes - the sources of evidence are very clear for all statements/’facts’ in the text output, AND the output enables you to link the source mentioned in the text to a full reference at the end of the publication or to another online source of information via hyperlink.
      1. Note: It is possible that a publication referring to a single source in both the text and the reference list/link will rate high on this question. I.e. the single source covers all included statements/’facts’ in the output.
3. **Is it clear when the information used or reported by the AI output was produced, and when the AI model was last updated?**
   1. **No - no dates have been given.**
   2. Partially - the date of the last AI model revision/update/amendment is clear on the webpage, OR dates for SOME but not ALL acknowledged sources have been given.
   3. Yes - dates for ALL acknowledged sources are clear, AND the date of the last AI model revision/update/amendment is clear on the webpage.
      1. Hints:
         - Dates of information sources (from question 2)
         - Print sources: dates for print sources will be found either with the references in the output or in the details of the references in the bibliography/reference list
         - Online sources: Dates for online sources such as webpages should be checked – copyright date, date of last revision/update/amendment. The date for a source such as email from a discussion group or a newsgroup is the posting date of the original message.
         - Date of AI model/version present (from ChatGPT): The date of the last revision, update or amendment to the output is clearly visible on the AI model website.
4. Is the AI output balanced and unbiased?
   1. 1: No - the information is completely unbalanced or biased.
   2. 2: Partially - some aspects of the information are unbalanced or biased.
   3. 3: Yes - the information is completely balanced and unbiased.
      1. Note:
         - Your rating should be based on your impression of the information as a whole.
      2. Hints:
         - Consider whether the information appears objective.
         - Consider whether there is evidence that a range of sources of information were used to compile the output, e.g. more than one research study or expert.
      3. Be wary if:
         - The output relies primarily on limited or single source
         - However, note that having a single source does not automatically infer bias.
         - the information is presented in a sensational, emotive or alarmist way.
5. Does the AI output provide details of additional sources of support or information?
   1. No - no additional sources of information are provided.
   2. Partially - the output provides details of additional source/s OR healthcare provider/organisation, however details are incomplete, OR refers user to their general healthcare provider without details.
      1. Incomplete details may include:
         - Incomplete reference.
         - Incomplete provider/ organisational contact details (if provided in output).
         - Non-functional hyperlink.
      2. If online sources are provided, they should be considered complete if they enable you to locate them on the internet easily e.g. the name of an organisation and its email address OR the website address (URL) either in full or as an external link.
   3. Yes - the output provides full details of source/s AND refers (you) to a healthcare provider or organisation for additional information/support, AND details are complete for at least one source/provider/organisation.
      1. Hint:
         - Look for suggestions for further reading (i.e. reference and/or hyperlink provided) or for details of other organisations providing advice and information about the condition and treatment choices.
         - A single reference may count as both an additional source of information AND referral to a healthcare provider/organisation if the provider/organisation is appropriate. Thus, there is an ability to score ‘3’ for a single reference to an external information source
6. Does the AI output refer to any areas of uncertainty?
   1. No - no uncertainty is mentioned.
   2. Partially – uncertainty is mentioned, but the information is unclear or incomplete.
   3. Yes - the output includes a clear reference to any uncertainty:
      1. This may be linked to specific statements/’facts’, or,
      2. May be covered in a more general aspect of the output discussion, or,
      3. May be related to a stated (in the output itself) limitation of the AI model, or,
      4. May be illustrated by deferral to a supporting information source (e.g. specific reference/publication/healthcare provider/healthcare organisation) or the users general health care provider.
7. Based on the answers to all of the above questions, rate the overall quality of the AI output as a source of information.
   1. Low - the output rated low (scores of 1) on the majority of questions.
      1. A low overall quality rating indicates the publication is ‘poor’ quality - it has serious shortcomings and is not a useful or appropriate source of information. It is unlikely to be of any benefit and should not be used.
   2. Moderate - the output rated high (score of 3) and low (score of 1) on a similar number of questions, OR the majority of questions rated in the mid-ranges (score of 2).
      1. A moderate overall quality rating indicates the output is ‘fair’ quality - it is a useful source of information but has some limitations. Additional information or support would definitely be needed.
   3. High - the publication rated high (score of 3) on the majority of questions.
      1. A high overall quality rating indicates the publication is ‘good’ quality - it is a useful and appropriate source of information.
