# Supplementary material for: Evaluating the Efficacy of ChatGPT as a Patient Education Tool in Prostate Cancer: Multimetric Assessment
Source: J Med Internet Res. 2024 Aug 14;26:e55939. doi: 10.2196/55939 (PMC11358656; doi:10.2196/55939)
Supplement: Multimedia Appendix 6 [file jmir_v26i1e55939_app6.docx]

1. Are the references real?
   - 1 - The references cited in the ChatGPT section do not exist or do not correlate with real-world references.
   - 2 - Only some of the references cited in the ChatGPT section exist OR the reference/s do correlate accurately with real-world references.
   - 3 - All of the references cited in the ChatGPT section exist and correlate with real-world references.
2. Do the references support the AI response?
   - 1 - The output of ChatGPT4 has little or no correlation with the references cited in the ChatGPT section.
   - 2 - The output of ChatGPT4 has moderate correlation with the references cited in the ChatGPT section, some inconsistencies or gaps may exist.
   - 3 - The output of ChatGPT4 has very high correlation with the references cited in the ChatGPT section, with no inconsistencies or gaps.
3. What are the sources of the references?
   - 1 - Not a source as per below
   - 2 - Resource developed or supplied by Government Organisation, Health Care Provider, Health Care Organisation.; and not related to those items in score of 3 below
   - 3 - Direct reference to scientific research article, guidelines of government or healthcare organisation.

ChatGPT Output with references:

**Q1). What are the symptoms of prostate cancer? Include references and quality of evidence.**

Chat GPT4 Output:

*Prostate cancer is a common type of cancer affecting the prostate gland in men. The symptoms of prostate cancer can vary, and in some cases, there may be no symptoms at all, particularly in the early stages. However, some common symptoms may include:*

1. *Frequent urination, especially at night*
2. *Difficulty starting or stopping urination*
3. *Weak or interrupted urine flow*
4. *Pain or burning sensation during urination*
5. *Blood in the urine or semen*
6. *Painful ejaculation*
7. *Pain in the lower back, hips, or upper thighs*
8. *Erectile dysfunction*

*It is important to note that these symptoms do not necessarily indicate prostate cancer and can also be caused by other prostate conditions, such as an enlarged prostate or prostatitis. It's essential to consult with a healthcare professional if you are experiencing any of these symptoms.*

*References:*

*American Cancer Society. (2021). Signs and Symptoms of Prostate Cancer. Retrieved from https://www.cancer.org/cancer/prostate-cancer/detection-diagnosis-staging/signs-symptoms.html [Quality of evidence: Authoritative source]*

*National Cancer Institute. (2021). Prostate Cancer - Symptoms and Signs. Retrieved from https://www.cancer.gov/types/prostate/patient/prostate-treatment-pdq#section/_46 [Quality of evidence: Authoritative source]*

*Prostate Cancer Foundation. (2021). Signs and Symptoms of Prostate Cancer. Retrieved from https://www.pcf.org/about-prostate-cancer/what-is-prostate-cancer/signs-and-symptoms-of-prostate-cancer/ [Quality of evidence: Authoritative source]*

Cancer Research UK. (2021). Symptoms of Prostate Cancer. Retrieved from https://www.cancerresearchuk.org/about-cancer/prostate-cancer/symptoms [Quality of evidence: Authoritative source]

**Q2). What are the risk factors for prostate cancer? Include references and quality of evidence.**

ChatGPT4 Output:

*Several factors can increase the risk of developing prostate cancer. While some risk factors are modifiable, others are not. The following are well-established risk factors for prostate cancer:*

1. *Age: The risk of developing prostate cancer increases with age, particularly after 50 years old. Most cases are diagnosed in men aged 65 or older.*
2. *Family history: Men with a close relative, such as a father or brother, who has had prostate cancer are at a higher risk of developing the disease themselves.*
3. *Race/ethnicity: Prostate cancer is more common among African American men and Caribbean men of African ancestry than in men of other races. The reasons for this disparity are not well understood.*
4. *Genetic factors: Certain inherited gene mutations, such as BRCA1 and BRCA2, can increase the risk of prostate cancer.*
5. *Diet: Some studies suggest that a diet high in red meat, processed meat, or high-fat dairy products may increase the risk of prostate cancer.*
6. *Obesity: Being obese may increase the risk of developing more aggressive or advanced prostate cancer.*

*References:*

*American Cancer Society. (2021). Risk Factors for Prostate Cancer. Retrieved from https://www.cancer.org/cancer/prostate-cancer/causes-risks-prevention/risk-factors.html [Quality of evidence: Authoritative source]*

*National Cancer Institute. (2021). Prostate Cancer - Risk Factors. Retrieved from https://www.cancer.gov/types/prostate/patient/prostate-treatment-pdq#section/_47 [Quality of evidence: Authoritative source]*

*Prostate Cancer Foundation. (2021). Risk Factors. Retrieved from https://www.pcf.org/about-prostate-cancer/what-is-prostate-cancer/risk-factors/ [Quality of evidence: Authoritative source]*

*Cancer Research UK. (2021). Risks and Causes of Prostate Cancer. Retrieved from https://www.cancerresearchuk.org/about-cancer/prostate-cancer/risks-causes [Quality of evidence: Authoritative source]*

*World Cancer Research Fund. (2018). Prostate Cancer. Retrieved from https://www.wcrf.org/dietandcancer/prostate-cancer/ [Quality of evidence: Authoritative source]*

**Q3). What is the survival rate of prostate cancer? Include references and quality of evidence.**

ChatGPT4 Output:

*The survival rate of prostate cancer is generally high, especially when detected early. Survival rates can be measured in different ways, such as the 5-year, 10-year, and 15-year survival rates, which represent the percentage of patients who live at least that long after diagnosis. It is important to remember that these rates are averages and can vary depending on individual factors such as age, overall health, and the stage of the cancer.*

*According to the American Cancer Society, the 5-year relative survival rate for all stages of prostate cancer combined is about 99%. The 10-year relative survival rate is 98%, and the 15-year relative survival rate is 96%. When the cancer is detected at a localized or regional stage (i.e., it has not spread beyond the prostate or has only spread to nearby areas), the 5-year relative survival rate is almost 100%.*

*However, for men with prostate cancer that has spread to distant parts of the body, the 5-year relative survival rate is about 30%. Despite this lower survival rate for advanced prostate cancer, new treatments are continuously being developed, which may improve the prognosis for individuals with metastatic disease.*

*References:*

*American Cancer Society. (2021). Survival Rates for Prostate Cancer. Retrieved from https://www.cancer.org/cancer/prostate-cancer/detection-diagnosis-staging/survival-rates.html [Quality of evidence: Authoritative source]*

*National Cancer Institute. (2021). Prostate Cancer - Survival Rates by Stage. Retrieved from https://www.cancer.gov/types/prostate/patient/prostate-treatment-pdq#section/_193 [Quality of evidence: Authoritative source]*

*Cancer Research UK. (2021). Prostate Cancer Survival Statistics. Retrieved from https://www.cancerresearchuk.org/health-professional/cancer-statistics/statistics-by-cancer-type/prostate-cancer/survival#heading-Zero [Quality of evidence: Authoritative source]*

**Q4). How is prostate cancer diagnosed? Include references and quality of evidence.**

ChatGPT4 Output:

*Prostate cancer is typically diagnosed through a combination of tests and examinations. The initial tests for prostate cancer detection may include:*

1. *Digital Rectal Exam (DRE): A healthcare professional inserts a gloved, lubricated finger into the rectum to feel the prostate for any irregularities in size, shape, or texture.*
2. *Prostate-Specific Antigen (PSA) blood test: This test measures the level of PSA, a protein produced by the prostate, in the blood. Elevated PSA levels can indicate prostate cancer, but other factors such as an enlarged prostate or inflammation can also cause high PSA levels.*

*If the results from the DRE and/or PSA tests indicate a potential issue, further tests may be conducted to confirm the diagnosis:*

1. *Imaging tests: These tests help determine if the cancer has spread beyond the prostate. Common imaging tests include transrectal ultrasound (TRUS), magnetic resonance imaging (MRI), computed tomography (CT) scan, and bone scans.*
2. *Biopsy: A prostate biopsy involves the removal of small tissue samples from the prostate gland, usually guided by a TRUS or MRI. The samples are then examined under a microscope by a pathologist to determine if cancer cells are present.*
3. *Gleason score and Grade Group: If cancer is detected in the biopsy samples, the pathologist will assign a Gleason score and a Grade Group based on the aggressiveness and appearance of the cancer cells. This information helps determine the appropriate treatment options.*

*It is important to consult with a healthcare professional if you have concerns about your prostate health or are experiencing symptoms that may indicate prostate cancer.*

*References:*

*American Cancer Society. (2021). Tests for Prostate Cancer. Retrieved from https://www.cancer.org/cancer/prostate-cancer/detection-diagnosis-staging/how-diagnosed.html [Quality of evidence: Authoritative source]*

*National Cancer Institute. (2021). Prostate Cancer - Diagnosis. Retrieved from https://www.cancer.gov/types/prostate/patient/prostate-treatment-pdq#section/_49 [Quality of evidence: Authoritative source]*

*Prostate Cancer Foundation. (2021). Diagnosing Prostate Cancer. Retrieved from https://www.pcf.org/about-prostate-cancer/what-is-prostate-cancer/diagnosing-prostate-cancer/ [Quality of evidence: Authoritative source]*

*Cancer Research UK. (2021). Tests for Prostate Cancer. Retrieved from https://www.cancerresearchuk.org/about-cancer/prostate-cancer/getting-diagnosed/tests [Quality of evidence: Authoritative source]*

**Q5). What age should men start getting screened for prostate cancer? Include references and quality of evidence.**

ChatGPT4 Output:

*The appropriate age for men to start getting screened for prostate cancer varies based on individual risk factors and professional guidelines. Different organizations have different recommendations:*

1. *American Cancer Society (ACS): The ACS recommends that men discuss the benefits, risks, and uncertainties of prostate cancer screening with their healthcare provider and make an informed decision. They suggest starting the conversation at:*

- *Age 50 for men with an average risk of prostate cancer who have at least a 10-year life expectancy.*
- *Age 45 for men with a high risk of prostate cancer, such as African American men and men with a first-degree relative (father or brother) diagnosed with prostate cancer before age 65.*
- *Age 40 for men with an even higher risk, such as those with more than one first-degree relative diagnosed with prostate cancer at an early age.*

1. *U.S. Preventive Services Task Force (USPSTF): The USPSTF recommends that men aged 55 to 69 discuss the potential benefits and harms of prostate cancer screening with their healthcare provider and make an individualized decision. For men aged 70 or older, the USPSTF recommends against routine PSA-based screening due to the potential harms outweighing the benefits.*

*It is essential for men to consult with their healthcare provider to determine the most appropriate age to start prostate cancer screening based on their individual risk factors and preferences.*

*References:*

*American Cancer Society. (2021). American Cancer Society Recommendations for Prostate Cancer Early Detection. Retrieved from https://www.cancer.org/cancer/prostate-cancer/detection-diagnosis-staging/acs-recommendations.html [Quality of evidence: Authoritative source]*

*U.S. Preventive Services Task Force. (2018). Final Recommendation Statement: Prostate Cancer: Screening. Retrieved from https://www.uspreventiveservicestaskforce.org/uspstf/recommendation/prostate-cancer-screening [Quality of evidence: Authoritative source]*

*Prostate Cancer Foundation. (2021). Screening Recommendations. Retrieved from https://www.pcf.org/about-prostate-cancer/what-is-prostate-cancer/screening-recommendations/ [Quality of evidence: Authoritative source]*

**Q6). What are the pros and cons of treatment options for prostate cancer? Include references and quality of evidence.**

ChatGPT4 Output:

*There are several treatment options for prostate cancer, and each has its pros and cons. The most appropriate treatment depends on factors such as the cancer stage, aggressiveness, patient's age, overall health, and personal preferences. Common treatment options include:*

1. *Active surveillance/watchful waiting*

| *Pros:*   - *Avoids side effects associated with other treatments* - *Postpones the need for more invasive treatments* - *Allows time for patients to consider other treatment options* | *Cons:*   - *Anxiety associated with not actively treating cancer* - *Possibility of cancer progression* - *Frequent monitoring and follow-up appointments* |
| --- | --- |

1. *Surgery (radical prostatectomy)*

| *Pros:*   - *Removes the entire prostate gland, which may offer a potential cure* - *Provides more accurate information about the cancer stage and grade* - *Reduces the need for further treatment if cancer is localized* | *Cons:*   - *Risk of complications, such as infection, bleeding, or blood clots* - *Potential side effects, such as urinary incontinence and erectile dysfunction* - *Recovery time from surgery* |
| --- | --- |

1. *Radiation therapy (external beam radiation therapy or brachytherapy)*

| *Pros:*   - *Non-invasive treatment (for external beam radiation therapy)* - *Can be used as the primary treatment or in conjunction with other treatments* - *Effective in treating localized or locally advanced prostate cancer* | *Cons:*   - *Side effects, such as urinary, bowel, or sexual problems* - *Fatigue during treatment* - *Multiple treatment sessions required (for external beam radiation therapy)* |
| --- | --- |

1. *Hormone therapy (androgen deprivation therapy)*

| *Pros:*   - *Slows the growth and spread of cancer cells* - *Can be used in conjunction with other treatments, such as radiation therapy* - *Effective in treating advanced or metastatic prostate cancer* | *Cons:*   - *Side effects, such as hot flashes, loss of libido, erectile dysfunction, bone loss, and metabolic changes* - *May not be effective indefinitely, as cancer cells can become resistant* - *Not a curative treatment* |
| --- | --- |

1. *Chemotherapy*

| *Pros:*   - *Can be effective in treating advanced or metastatic prostate cancer* - *May be used when hormone therapy is no longer effective* | *Cons:*   - *Side effects, such as nausea, vomiting, hair loss, fatigue, and increased risk of infection* - *Not a curative treatment* |
| --- | --- |

1. *Immunotherapy*

| *Pros:*   - *Uses the patient's immune system to fight cancer* - *Can be effective for some men with advanced or metastatic prostate cancer* | *Cons:*   - *Not effective for all patients* - *Side effects, such as flu-like symptoms, fatigue, and infusion reactions* |
| --- | --- |

*It is essential for patients to discuss the pros and cons of each treatment option with their healthcare team to determine the most suitable approach for their specific situation.*

***References:***

*American Cancer Society. (2021). Treatment Choices for Prostate Cancer, by Stage. Retrieved from https://www.cancer.org/cancer/prostate-cancer/treating/by-stage.html [Quality of evidence: Authoritative source]*

*National Cancer Institute. (2021). Prostate Cancer Treatment (PDQ®)–Patient Version. Retrieved from https://www.cancer.gov/types/prostate/patient/prostate-treatment-pdq [Quality of evidence: Authoritative source]*

*Prostate Cancer Foundation. (2021). Prostate Cancer Treatment Options. Retrieved from https://www.pcf.org/about-prostate-cancer/prostate-cancer-treatment/ [Quality of evidence: Authoritative source]*

*Cancer Research UK. (2021). Treatment Choices for Prostate Cancer. Retrieved from https://www.cancerresearchuk.org/about-cancer/prostate-cancer/treatment/which-treatment [Quality of evidence:*

**Q7). How does prostate cancer affect sexual function? Include references and quality of evidence.**

ChatGPT4 Output:

*Prostate cancer and its treatments can have a significant impact on sexual function. The most common sexual side effects experienced by men with prostate cancer are erectile dysfunction (ED) and changes in libido. The extent of these side effects depends on factors such as the type and stage of cancer, the treatment received, and the patient's age and overall health.*

1. *Erectile dysfunction (ED): The inability to achieve or maintain an erection sufficient for sexual intercourse can occur due to prostate cancer treatments such as surgery, radiation therapy, or hormone therapy.*

- *Surgery (radical prostatectomy) can damage the nerves and blood vessels responsible for erections. Nerve-sparing surgical techniques may reduce the risk of ED, but it's not always possible to preserve the nerves.*
- *Radiation therapy can cause ED by damaging the nerves and blood vessels in the area. The risk of ED may increase over time following radiation therapy.*
- *Hormone therapy can lower testosterone levels, leading to decreased libido and ED.*

1. *Changes in libido: A reduced interest in sex can occur due to hormone therapy, which lowers testosterone levels. Additionally, anxiety and stress associated with a cancer diagnosis and treatment may also contribute to changes in libido.*

*It's important to discuss any concerns about sexual function with a healthcare provider, as they can recommend various strategies and treatments to help manage these side effects. These may include medications, penile implants, vacuum erection devices, or counseling.*

*References:*

*American Cancer Society. (2021). Sexual Side Effects of Cancer Treatment. Retrieved from https://www.cancer.org/treatment/treatments-and-side-effects/physical-side-effects/fertility-and-sexual-side-effects/sexuality-for-men/sexual-side-effects.html [Quality of evidence: Authoritative source]*

*Prostate Cancer Foundation. (2021). Side Effects of Prostate Cancer Treatment. Retrieved from https://www.pcf.org/about-prostate-cancer/prostate-cancer-side-effects/ [Quality of evidence: Authoritative source]*

*National Cancer Institute. (2021). Prostate Cancer Treatment (PDQ®)–Patient Version. Retrieved from https://www.cancer.gov/types/prostate/patient/prostate-treatment-pdq#section/_185 [Quality of evidence: Authoritative source]*

*Cancer Research UK. (2021). Sex and Prostate Cancer. Retrieved from https://www.cancerresearchuk.org/about-cancer/prostate-cancer/living-with/sex [Quality of evidence: Authoritative source]*

**Q8). How does prostate cancer affect bladder function? Include references and quality of evidence.**

ChatGPT4 Output:

*Prostate cancer and its treatments can have a significant impact on bladder function. The most common bladder-related side effects are urinary incontinence and urinary irritation. The extent of these side effects depends on factors such as the type and stage of cancer, the treatment received, and the patient's age and overall health.*

1. *Urinary incontinence: The involuntary leakage of urine can occur due to prostate cancer treatments, such as surgery or radiation therapy.*

- *Surgery (radical prostatectomy) can weaken the muscles that control urine flow, leading to stress incontinence (leakage during physical activities) or urge incontinence (sudden need to urinate). In most cases, incontinence improves over time, but some men may continue to experience long-term issues.*
- *Radiation therapy can cause irritation to the bladder and urethra, leading to temporary or long-term incontinence.*

1. *Urinary irritation: Symptoms such as frequent urination, urgent need to urinate, pain or burning during urination, and difficulty starting or stopping urine flow can occur due to prostate cancer treatments, such as radiation therapy or hormone therapy. These symptoms are often temporary and may improve after treatment is completed.*

*It's important to discuss any concerns about bladder function with a healthcare provider, as they can recommend various strategies and treatments to help manage these side effects. These may include pelvic floor exercises, medications, bladder training, or surgical interventions.*

*References:*

*American Cancer Society. (2021). Urinary Side Effects of Cancer Treatment. Retrieved from https://www.cancer.org/treatment/treatments-and-side-effects/physical-side-effects/fertility-and-sexual-side-effects/sexuality-for-men/urinary-side-effects.html [Quality of evidence: Authoritative source]*

*Prostate Cancer Foundation. (2021). Side Effects of Prostate Cancer Treatment. Retrieved from https://www.pcf.org/about-prostate-cancer/prostate-cancer-side-effects/ [Quality of evidence: Authoritative source]*

*National Cancer Institute. (2021). Prostate Cancer Treatment (PDQ®)–Patient Version. Retrieved from https://www.cancer.gov/types/prostate/patient/prostate-treatment-pdq#section/_185 [Quality of evidence: Authoritative source]*

*Cancer Research UK. (2021). Urinary Problems and Prostate Cancer. Retrieved from https://www.cancerresearchuk.org/about-cancer/prostate-cancer/living-with/urinary-problems [Quality of evidence: Authoritative source]*
